# Transcriptomic analysis of *Campylobacter jejuni* NCTC 11168 in response to epinephrine and norepinephrine

**DOI:** 10.3389/fmicb.2015.00452

**Published:** 2015-05-18

**Authors:** Fuzhou Xu, Cun Wu, Fangfang Guo, Guolin Cui, Ximin Zeng, Bing Yang, Jun Lin

**Affiliations:** ^1^Beijing Key Laboratory for Prevention and Control of Infectious Diseases in Livestock and Poultry, Institute of Animal Husbandry and Veterinary Medicine, Beijing Academy of Agriculture and Forestry SciencesBeijing, China; ^2^Department of Animal Science, The University of TennesseeKnoxville, TN, USA

**Keywords:** *Campylobacter jejuni*, catecholamine hormones, microarray, growth promotion, virulence, adhesion and invasion

## Abstract

Upon colonization in the host gastrointestinal tract, the enteric bacterial pathogen *Campylobacter jejuni* is exposed to a variety of signaling molecules including the catecholamine hormones epinephrine (Epi) and norepinephrine (NE). NE has been observed to stimulate the growth and potentially enhance the pathogenicity of *C. jejuni*. However, the underlying mechanisms are still largely unknown. In this study, both Epi and NE were also observed to promote *C. jejuni* growth in MEMα-based iron-restricted medium. Adhesion and invasion of Caco-2 cells by *C. jejuni* were also enhanced upon exposure to Epi or NE. To further examine the effect of Epi or NE on the pathobiology of *C. jejuni*, transcriptomic profiles were conducted for *C. jejuni* NCTC 11168 that was cultured in iron-restricted medium supplemented with Epi or NE. Compared to the genes expressed in the absence of the catecholamine hormones, 183 and 156 genes were differentially expressed in *C. jejuni* NCTC 11168 that was grown in the presence of Epi and NE, respectively. Of these differentially expressed genes, 102 genes were common for both Epi and NE treatments. The genes differentially expressed by Epi or NE are involved in diverse cellular functions including iron uptake, motility, virulence, oxidative stress response, nitrosative stress tolerance, enzyme metabolism, DNA repair and metabolism and ribosomal protein biosynthesis. The transcriptome analysis indicated that Epi and NE have similar effects on the gene expression of *C. jejuni*, and provided insights into the delicate interaction between *C. jejuni* and intestinal stress hormones in the host.

## Introduction

Since the late 1970s, *Campylobacter jejuni* has emerged as the leading bacterial cause of foodborne human diseases in many industrialized countries (Olson et al., 2008). This pathogenic organism causes watery diarrhea and/or hemorrhagic colitis in humans and is also associated with Guillain-Barre syndrome, an acute flaccid paralysis that may lead to respiratory muscle compromise and death (Nachamkin et al., 1998). In contrast to its increased prevalence, limited progress has been made in the development of effective intervention strategies against *Campylobacter* infections in humans and animal reservoirs (Lin, 2009), which is primarily due to lack of good understanding of *Campylobacter* pathogenesis.

Through oral ingestion, *C. jejuni* enters the host intestine via the stomach acid barrier and colonizes the distal ileum and cecum. Once inside the intestine, *C. jejuni* is faced with various compounds and multiple levels of stresses, which may serve as cues to modulate *C. jejuni* gene expression for successful colonization in the intestine (Everest, 2007). Recently, gut catecholamines, a group of host stress hormones that could be released by the local enteric nervous system, have been reported to play an important role in host-bacteria communication in the intestine (Everest, 2007; Freestone, 2013). Specifically, catecholamines, such as epinephrine (Epi) and norepinephrine (NE), can modulate bacterial infectivity in different ways, such as promoting bacterial growth, stimulating virulence gene expression, and enhancing attachment to host tissue (Freestone et al., 2008; Lyte et al., 2011; Freestone, 2013). These unique findings have led to a new multidisciplinary research area called “Microbial Endocrinology” (Lyte, 1993, 2004; Freestone et al., 2008).

Recently, the catecholamine hormone NE was also observed to increase the virulence-associated properties of *C. jejuni* (Cogan et al., 2007) and stimulate the growth of *C. jejuni* under iron-limited condition (Cogan et al., 2007; Zeng et al., 2009; Xu et al., 2010). However, the underlying mechanisms of NE-mediated growth promotion and virulence enhancement in *C. jejuni* are still unknown. To address this issue, in this study, we used microarray to analyze transcriptomic profile of *C. jejuni* NCTC 11168 in response to catecholamine hormones Epi or NE under iron-restricted condition. The findings from this study provide a foundation for us to elucidate molecular basis of *C. jejuni* response to gut catecholamines in the future.

## Materials and methods

### Bacterial strain and culture conditions

*C. jejuni* NCTC 11168 was routinely grown in Mueller-Hinton (MH) broth (Difco, Sparks, MD) or on agar at 42°C in a microaerophilic incubator (5% O_2_, 10% CO_2_, 85% N_2_). To assess the effects of Epi or NE on the bacteria, *C. jejuni* NCTC 11168 was grown in the iron-restricted minimal essential medium alpha (MEMα) (Invitrogen) supplemented with 10% fetal bovine serum (FBS) (Hyclone).

### Bacterial growth assay

The effects of Epi and NE on the growth of *C. jejuni* NCTC 11168 were assessed using standard bacterial growth assay. Briefly, *Campylobacter* cells were grown in MH broth to log phase and were then pelleted, washed once and resuspended in MEMα medium. The bacterial suspension was inoculated into MEMα medium at a final concentration of 1 × 10^2^ cfu/ml with or without 100 μM of (−)-epinephrine (+)-bitartrate salt (Epi) or L-(−)-norepinephrine (+)-bitartrate salt monohydrate (NE) (Sigma-Aldrich). Cultures were grown in 6-well plates with 5 ml of culture per well for 48 h. The optical density of each well at 600 nm was determined every 12 h of incubation using Eppendorf BioPhotometer. All assays were performed in three independent experiments with triplicate setting for each experiment. Results represent the mean ± the standard deviation (SD) of three independent experiments. The paired Student's *t*-test was used for statistical analysis at 5% level of significance.

### Adhesion and invasion assay of Caco-2 cells

The ability of *Campylobacter* to adhere and invade Caco-2 cells was assessed using assays as described in previous publications (Cogan et al., 2007; Ganan et al., 2010) with some modifications. Briefly, Caco-2 cells were grown and maintained in DMEM (Invitrogen) containing 1% non-essential amino acids and 10% fetal bovine serum. The Caco-2 cells were seeded into 24-well plates at 1 × 10^5^ cells/well and incubated at 37°C in a 5% CO_2_ atmosphere until confluence. The *C. jejuni* NCTC 11168 was grown in MEMα medium in the presence or absence of Epi or NE for 36 h (mid-log phase) under microaerophilic condition as described above. The *C. jejuni* cells were pelleted, washed three times with MEMα medium, and finally resuspended in MEMα medium. The *C. jejuni* inoculum was added into triplicate wells of confluent monolayer of Caco-2 cells to give a MOI of 100:1. Following incubation at 37°C for 2 h, the non-adherent bacteria were removed by washing four times with PBS. To determine bacterial adherence, the cell monolayer were lysed with 1% Triton X-100 (Sigma-Aldrich) and total intracellular and extracellular bacteria were enumerated by serial dilutions. To determine bacterial invasion, the cell monolayer was incubated in fresh PBS containing 1% FBS and 150 μg/ml gentamicin for 2 h to kill remaining viable extracellular bacteria. Then the cell monolayer was washed three times with PBS and lysed with 1% Triton X-100 to determine the intracellular bacteria by serial dilutions. Results were expressed as percent of adhered or invasive bacteria relative to inoculum as described in previous study (Ganan et al., 2010). The experiments were repeated three times, and the results shown are the mean ± the standard deviation (SD). The paired Student's *t*-test was used for statistical analysis at 5% level of significance.

### Microarray protocol

The microarray slides for *C. jejuni* NCTC 11168 were designed using Agilent eArray program and manufactured by Agilent Technologies. Each customized microarray contained spots in 8 replicates with 1683 60-mer oligonucleotides specifically targeting the 1683 protein-coding genes in *C. jejuni* NCTC 11168 genome (Parkhill et al., 2000; Gundogdu et al., 2007).

As described in the bacterial growth assay, the *C. jejuni* NCTC 11168 cells harvested from MH broth were inoculated into MEMα medium at a final concentration of 1 × 10^2^ cfu/ml with or without 100 μM Epi or NE. The *Campylobacter* cells were grown in 6-well plates with 5 ml per well at 42°C under microaerophilic condition. At the mid-log phase (approximately 36 h of growth), the Epi or NE treated and untreated *C. jejuni* NCTC 11168 cultures were collected and treated with RNA*later* stabilization solution (Ambion, Life Technologies). Three (for Epi or NE treated cultures) or four (for untreated control cultures) independent biological replicates were performed. The harvested *C. jejuni* cells were pelleted to remove the RNA*later* solution and the cell pellets were used for RNA extraction using RiboPure™-Bacteria Kit (Ambion, Life Technologies) according to the manufacturer's instruction. The quality and quantity of total RNA were determined by an Agilent Bioanalyzer 2100 (Agilent technologies, Santa Clara, CA, US). The extracted RNA was further purified by RNeasy micro kit and RNase-Free DNase Set (QIAGEN, GmBH, Germany). Total RNA was amplified and labeled by Low Input Quick Amp Labeling Kit, One-Color (Agilent technologies, Santa Clara, CA, US) by following the manufacturer's instructions. The labeled cRNA were purified by RNeasy mini kit (QIAGEN, GmBH, Germany). Each slide was hybridized with 600 ng of Cy3-labeled cRNA using Gene Expression Hybridization Kit (Agilent technologies, Santa Clara, CA, US). Following 17 h of hybridization, the slides were washed in staining dishes with Gene Expression Wash Buffer Kit (Agilent technologies, Santa Clara, CA, US). Slides were scanned by Agilent Microarray Scanner (Agilent technologies, Santa Clara, CA, US) with default settings, dye channel: green, scan resolution = 5 μm, PMT 100%, 10%, 16 bit.

The microarray data were collected with Feature Extraction software 10.7 (Agilent technologies). Raw data were normalized by Quantile algorithm, Gene Spring Software 11.0 (Agilent technologies). All the data are MIAME compliant and the raw data has been deposited in the NCBI GEO database under the number GSE65187. Subsequently, the normalized data were statistically analyzed using a mixed-linear model with the SAS statistical package, accounting for fixed effects of treatment and random effects of replications, samples, and slides. *P*-values were adjusted by a False Discovery Rate of 5% and differential expression declared when *P* < 0.05. For this study, we chose *P* < 0.05 and fold change equal to or greater than 1.5 as the cutoff for significant differential expression between different growth conditions.

The differentially expressed genes were categorized by their Clusters of Orthologous Groups (COG) annotations (Tatusov et al., 2003). Genes with no COG annotation were included in the poorly characterized category. Moreover, the differentially expressed genes of *C. jejuni* following exposure to Epi or NE were analyzed and compared with two previously published datasets which obtained using microarray (Palyada et al., 2004) or RNA-Seq (Butcher and Stintzi, 2013) approaches under iron replete and iron limited conditions.

### Real-time quantitative RT-PCR

Total 10 genes were selected from microarray data analysis and validated by qRT-PCR analysis. The selected genes and the primers used for qRT-PCR are listed in Table S1. Briefly, RNA was extracted from the treatment and control cultures as described above. The qRT-PCR was performed using iQ SYBR green Supermix kit (Bio-Rad) with the IQ5 Real-time-PCR Detection System (Bio-Rad). The qRT-PCR parameters consisted of a cDNA synthesis at 50°C for 10 min and reverse transcriptase inactivation at 95°C for 5 min, followed by PCR cycling and detection of 95°C for 10 s and 60°C for 30 s (40 cycles). Similar to a previous study by which the relative expression level of each gene was normalized to the invariant expression genes *slyD* or *ilvC* (Palyada et al., 2004), the relative transcription level of each gene in this study was normalized to the *Cj0131* gene which displayed no changes under different growth conditions. Quantitative values were obtained with the comparative threshold cycle (ΔΔC_T_) method. The transcript level from each RNA sample was assayed three times, and the mean C_T_ value used for further analysis. The difference in the transcription of each specific gene was calculated by the 2^−ΔΔCT^ method.

## Results

### Both Epi and NE promote the growth of *C. jejuni*

**Figure 1:**
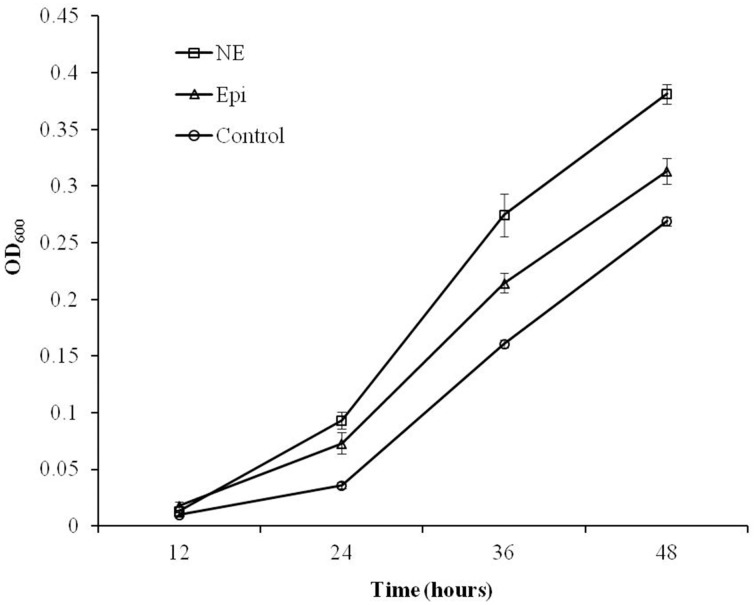
**Growth response of *C. jejuni* NCTC 11168 to Epi or NE in iron-restricted MEMα medium containing 10% FBS**. Results represent the mean plus the standard deviation (SD) of three independent experiments.

As shown in Figure 1, the growth of *C. jejuni* in the presence of Epi or NE was significantly enhanced compared with control culture at all tested time points (*P* < 0.01). By 24 h of growth, the addition of Epi or NE resulted in 3 and 2 folds increase in growth (reflected by OD_600_), respectively. The growth assay also showed that the growth rate of *C. jejuni* was higher in NE-supplemented culture than in Epi-supplemented culture (*P* < 0.01).

### Enhancement of adhesion and invasion of Caco-2 cells by Epi and NE

**Figure 2:**
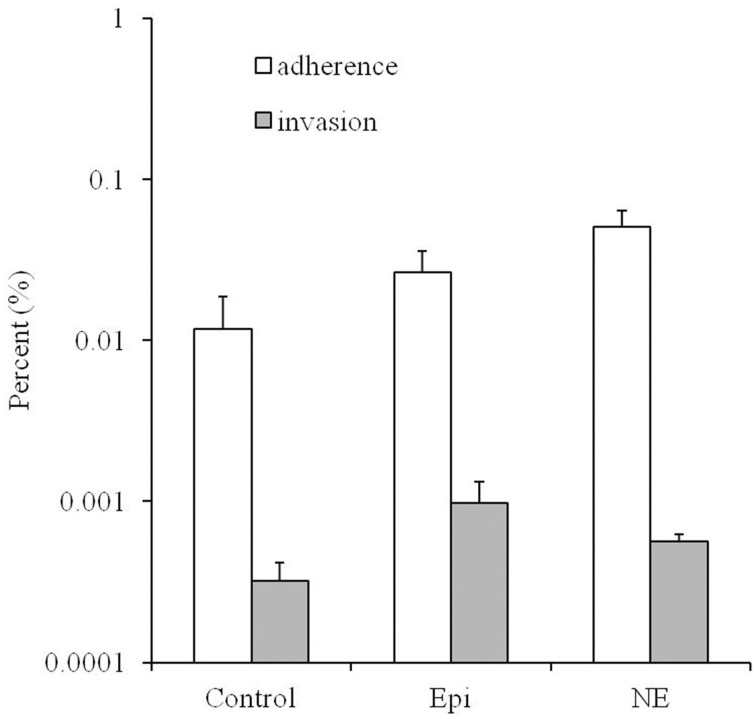
**Effects of Epi and NE on adhesion and invasion of Caco-2 cells by *C. jejuni* NCTC 11168**. Results were expressed as percent of adhered or invasive bacteria relative to inoculum. Results represent the mean plus the standard deviation (SD) of three independent experiments.

As showed in Figure 2, the adherence and invasion rates for the *Campylobacter* treated by Epi or NE were significantly higher than the untreated control group (*P* < 0.01). Specifically, the adherence rates are 0.012, 0.026, 0.051% for control, Epi, and NE group, respectively while invasion rates are 0.00032, 0.00098, 0.00056% for control, Epi, and NE group, respectively. Moreover, NE treated bacteria showed significantly higher adhesive (*P* < 0.01) and lower invasive (*P* < 0.05) capacities than bacteria treated by Epi.

### Effects of Epi or NE on the transcriptome of *C. jejuni*

To investigate the transcriptional profiles of *C. jejuni* NCTC 11168 in response to Epi or NE, microarrays were used to compare the transcriptome of the *C. jejuni* grown in Epi or NE-supplemented MEMα medium to those grown in the absence of the catecholamines. The genes whose expression was altered by at least 1.5-fold (*P* < 0.05) were designated as differentially expressed as described in previous studies (Dowd, 2007; Li et al., 2012). The numbers of genes differentially expressed upon exposure to Epi or NE were calculated and analyzed (Table 1). A total of 183 (96 up-regulated genes and 87 down-regulated genes) and 156 genes (86 up-regulated genes and 70 down-regulated genes) were differentially expressed by Epi and NE, respectively (Tables S3, S4), with 102 differentially expressed genes (56 up-regulated genes and 46 down-regulated genes) common between the two treatments (Tables S3, S4).

**Table 1:** **Summary of differentially expressed genes in *Campylobacter jejuni* NCTC 11168 upon exposure to Epi or NE**.

**Genes regulated by**	**No. of genes**		**Total**
	**Up-regulated**	**Down-regulated**	
Epi	86	70	156
NE	96	87	183
Epi + NE^a^	56	46	102
Epi only^b^	30	24	54
NE only^c^	40	41	81

a *Differentially expressed genes that were commonly identified upon Epi and NE treatments*.

b *Differentially expressed genes were only identified upon Epi treatment*.

c *Differentially expressed genes were only identified upon NE treatment*.

To validate the microarray data, the expression of 10 genes was selected and confirmed by qRT-PCR. As shown in Table 2, the qRT-PCR results are consistent with the data from microarray, demonstrating the reliability of the microarray data.

**Table 2:** **Validation of microarray data by qRT-PCR**.

**Gene**	**Fold change by Epi**		**Fold change by NE**	
	**Microarray**	**qRT-PCR^a^**	**Microarray**	**qRT-PCR^a^**
*flgL*	2.51	1.49	2.99	1.59
*flgH*	2.98	1.91	4.21	2.39
*Cj1729c*	2.90	2.25	3.25	1.80
*fdxA*	3.50	2.43	2.67	1.28
*Cj0037c*	4.00	1.87	6.29	2.97
*chuB*	−16.43	−13.00	−8.79	−13.64
*cfrA*	−3.37	−4.92	−2.81	−4.96
*exbB1*	−3.24	−6.63	−2.96	−7.36
*Cj0178*	−3.10	−5.59	−2.50	−6.75
*Cj0131*	1.01	1.00	1.02	1.00

a *Mean of three independent experiments*.

**Figure 3:**
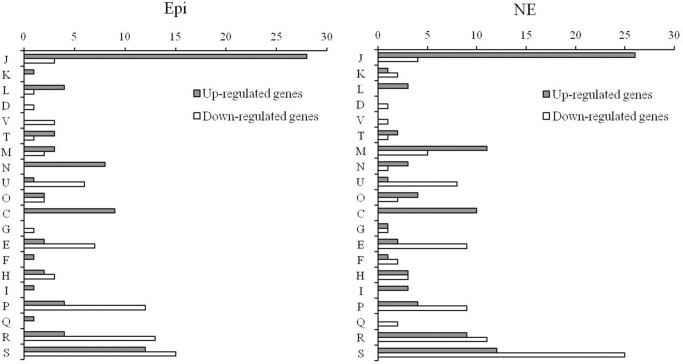
**Functional classification of differentially expressed genes in *C. jejuni* NCTC 11168 upon exposure to Epi or NE**. Gene functions are sorted according to COG categories: Information storage and processing (J: Translation, ribosomal structure, and biogenesis; K: Transcription; L: Replication, recombination and repair); Cellular processes and signaling (D: Cell cycle control, cell division, chromosome partitioning; V: Defense mechanisms; T: Signal transduction mechanisms; M: Cell wall/membrane/envelope biogenesis; N: Cell motility; U: Intracellular trafficking, secretion, and vesicular transport; O: Posttranslational modification, protein turnover, chaperones); Metabolism (C: Energy production and conversion; G: Carbohydrate transport and metabolism; E: Amino acid transport and metabolism; F: Nucleotide transport and metabolism; H: Coenzyme transport and metabolism; I: Lipid transport and metabolism; P: Inorganic ion transport and metabolism; Q: Secondary metabolites biosynthesis, transport and catabolism); Poorly characterized (R: General function prediction only; S: Function unknown or not in COG database).

The individual genes that were found to be differentially expressed in response to Epi or NE were sorted by COG category. The differentially expressed genes for Epi or NE treatment were classified into 20 different functional categories respectively (Figure 3). Among differentially expressed genes by Epi, approximately 28% genes are poorly characterized (R+S); the main COG categories were J (20%), P (10.3%), E (5.8%), C (5.8%), N (5.1%), and U (4.5%). For differentially expressed genes by NE, in addition to the poorly characterized (R+S) genes (31%), the main COG categories include J (16%), M (8.7%), P (7.1%), E (6%), C (5.5%), and U (4.9%). There is only slight difference observed between Epi-regulated and NE-regulated genes. The wide diversity in functional categories suggests that Epi or NE treatment has a significant influence on metabolism, transcription and translation, cellular processes and signaling of *C. jejuni* NCTC 11168.

Based on the COG category, the majority of differentially expressed genes in response to Epi or NE can be further classified into several main functional groups as described below.

#### Iron uptake systems

As shown in Table 3, the genes down-regulated by both Epi and NE include almost all the known iron uptake transporters in *C. jejuni*, including the ferric enterobactin receptor (*cfrA*), rhodotorulic acid (*Cj1658, Cj1660-1662*), hemin (*chuABCD*), and transferrin (*cfbpABC, Cj0176c-0178*) (Miller et al., 2009). Interestingly, no significant differential expression was observed for genes belonging to ABC transport system *ceuBCDE* and the rhodotorulic acid transporter *p19*. In addition, the genes belong to the part of energy transduction systems (*exbB1-exbD1*, *exbB2-exbD2*) were down-regulated by Epi or NE while the expression of *tonB1*, *tonB2*, *tonB3*, and *exbB3-exbD3* was not affected by Epi or NE. Expression of the ferric uptake regulator Fur was not affected by Epi or NE either.

**Table 3:** **Fold change of iron-uptake system components in *Campylobacter jejuni* upon exposure to Epi or NE**.

**Gene ID**	**Gene symbol**	Fold change^*^		**Description**
		**Epi**	**NE**	
**FERRIC-ENTEROBACTIN**				
*Cj0755*	*cfrA*	**−3.4**	**−2.8**	Ferric enterobactin uptake receptor
*Cj1352*	*ceuB*	1.1	1.2	Enterobactin uptake permease
*Cj1353*	*ceuC*	−1.2	−1.4	Enterobactin uptake permease
*Cj1354*	*ceuD*	1.0	−1.2	Enterobactin uptake ATP-binding protein
*Cj1355*	*ceuE*	1.3	1.4	Enterobactin uptake substrate-binding protein
**RHODOTORULIC ACID**				
*Cj1658*	*Cj1658*	**−1.9**	**−2.0**	Iron permease
*Cj1659*	*p19*	−1.3	−1.3	Hypothetical protein
*Cj1660*	*Cj1660*	**−1.7**	**−1.6**	Integral membrane protein
*Cj1661*	*Cj1661*	**−1.5**	**−1.5**	ABC transporter permease
*Cj1662*	*Cj1662*	**−1.5**	**−1.6**	Integral membrane protein
*Cj1663*	*Cj1663*	−1.3	**−1.6**	ABC transporter ATP-binding protein
**HEMIN**				
*Cj1613c*	*chuZ*	1.2	1.1	Putative pyridoxamine 5′-phosphate oxidase
*Cj1614*	*chuA*	**−2.3**	**−1.8**	Hemin uptake system outer membrane receptor
*Cj1615*	*chuB*	**−16.4**	**−8.8**	Hemin uptake system permease
*Cj1616*	*chuC*	**−6.1**	**−4.1**	Hemin uptake system ATP-binding protein
*Cj1617*	*chuD*	**−1.7**	**−1.6**	Hemin uptake system substrate-binding protein
**TRANSFERRIN**				
*Cj0173c*	*cfbpC*	−1.4	−1.5	Iron-uptake ABC transporter ATP-binding protein
*Cj0174c*	*cfbpB*	**−2.3**	**−2.1**	Iron-uptake ABC transporter permease
*Cj0175c*	*cfbpA*	**−2.2**	**−1.7**	Iron-uptake ABC transporter substrate-binding protein
*Cj0176c*	*Cj0176c*	**−2.5**	**−2.2**	Lipoprotein
*Cj0177*	*Cj0177*	**−6.1**	**−6.2**	Iron transport protein
*Cj0178*	*Cj0178*	**−3.1**	**−2.5**	TonB-denpendent outer membrane receptor
**ENERGY TRANSDUCTION SYSTEMS**				
*Cj0179*	*exbB1*	**−3.2**	**−3.0**	Biopolymer transport protein
*Cj0180*	*exbD1*	**−4.7**	**−4.0**	Biopolymer transport protein
*Cj0181*	*tonB1*	−1.3	−1.1	TonB transport protein
*Cj1628*	*exbB2*	**−2.9**	**−2.6**	Putative exbB/tolQ family transport protein
*Cj1629*	*exbD2*	**−3.1**	**−2.9**	Putative exbD/tolR family transport protein
*Cj1630*	*tonB2*	−1.3	−1.2	TonB transport protein
*Cj0109*	*exbB3*	−1.3	−1.5	Putative MotA/TolQ/ExbB proton channel family protein
*Cj0110*	*exbD3*	−1.0	−1.3	Putative exbD/tolR family transport protein
*Cj0753c*	*tonB3*	−1.5	−1.3	TonB transport protein
**FERRIC UPTAKE REGULATOR**				
*Cj0400*	*fur*	1.1	1.0	Ferric uptake regulator protein

* *Significant difference of fold changes is shown in boldface*.

#### Bacterial motility (flagellar assembly pathway)

Many of the up-regulated genes are involved in flagellar assembly pathway (Tables S3, S4). In the presence of Epi or NE, the flagellar genes involved into the flagellar biogenesis were up-regulated, which include the genes encoding hook-filament junction (*flgL*), hook (*flgE* and *flgE2*), FlgE chaperones (*flgD* and *fliK*), and rod (*flgG2, flgH*, and *flgI*) (Table 4). No difference was observed for the expression of the genes encoding other flagellar components including filament (*flaA, flaG*, and *fliD*), rod (*fliE, fliF, fliG*, and *fliH*) and regulatory proteins (*fliS, flgM*, *flgR*, and *fliA*) (Table 4). Chemotaxis also plays an essential role in flagella locomotion and bacterial motility in the presence of specific chemoeffectors (Zautner et al., 2012). The genes encoding chemotaxis histidine kinase CheA (*Cj0284c*) and the coupling scaffold protein CheW (*Cj0283c*) were both up-regulated with 2.1 and 1.8-fold by Epi, respectively. Expression of CheA and CheW genes was also increased about 2.4 and 2.1-fold by NE, respectively.

**Table 4:** **Fold change of the flagella assembly components in *Campylobacter jejuni* upon exposure to Epi or NE**.

**Gene ID**	**Gene symbol**	Fold change^*^		**Description**
		**Epi**	**NE**	
**FILAMENT**				
*Cj1339c*	*flaA*	1.2	1.4	Flagellin
*Cj0547*	*flaG*	1.1	1.2	Flagellar protein
*Cj0548*	*fliD*	1.1	1.2	Flagellar capping protein
**HOOK-FILAMENT JUNCTION**				
*Cj0887c*	*flgL*	**2.5**	**3.0**	Flagellar hook-associated protein
*Cj1466*	*flgK*	1.5	1.4	Flagellar hook-associated protein
**HOOK**				
*Cj0043*	*flgE*	**3.0**	**8.1**	Flagellar hook protein
*Cj1729c*	*flgE2*	**2.9**	**8.9**	Flagellar hook protein
**FlgE CHAPERONES**				
*Cj0042*	*flgD*	**4.5**	**4.8**	Flagellar basal body rod modification protein
*Cj0041*	*fliK*	**2.1**	**2.7**	Flagellar hook-length control protein
**ROD**				
*Cj0698*	*flgG*	1.2	1.4	Flagellar basal body rod protein
*Cj0697*	*flgG2*	**1.7**	**2.0**	Flagellar basal-body rod protein
*Cj0687c*	*flgH*	**3.0**	**4.2**	Flagellar basal body L-ring protein
*Cj1462*	*flgI*	**3.5**	**4.9**	Flagellar basal body P-ring protein
*Cj0526c*	*fliE*	1.0	−1.2	Flagellar hook-basal body protein
*Cj0318*	*fliF*	1.2	1.3	Flagellar MS-ring protein
*Cj0319*	*fliG*	1.2	1.3	Flagellar motor switch protein G
*Cj0320*	*fliH*	1.0	1.0	Flagellar assembly protein H
*Cj1179c*	*fliR*	−1.3	−**1.5**	Flagellar biosynthesis protein
*Cj0820c*	*fliP*	1.3	**1.7**	Flagellar biosynthesis protein
**REGULATORS**				
*Cj0549*	*fliS*	1.0	1.0	Flagellar protein
*Cj1464*	*flgM*	1.1	1.1	Antisigma factor
*Cj1024c*	*flgR*	1.2	1.2	Sigma-54 associated transcriptional activator
*Cj0061c*	*fliA*	1.0	1.1	Flagellar biosynthesis sigma factor

* *Significant difference of fold changes is shown in boldface*.

#### Oxidative stress responses

Production of reactive oxygen species (ROS) during normal metabolic processes is detrimental to *C. jejuni*. Therefore, *C. jejuni* has evolved defense mechanisms to protect it under oxidative stress. In this study, genes of *katA* and *Cj1386* were observed to be down-regulated by the catecholamines (2.8 and 1.7-fold by Epi; 2.9 and 1.8-fold by NE). The genes of a non-haem iron protein Rrc (*Cj0012c*) and ferredoxin FdxA (*Cj0333c*) were up-regulated by Epi or NE. The hemerythrin HerB (*Cj1224*) involved in protecting the activity of oxidoreductases from oxygen damage (Kendall et al., 2014) was also up-regulated by Epi.

#### Nitrosative stress tolerance

The anaerobic-respiration pathways are very important in *C. jejuni* growth and colonization (Weingarten et al., 2008). The periplasmic nitrite reductase NrfH-NrfA (*Cj1358c-Cj1357c*), the nitrate reductase NapA/B (*Cj0780/Cj0783*) and the *cbb3*-terminal oxidase *ccoNOQ* operon (*Cj1490c*-*Cj1488c*) were up-regulated by Epi or NE, which indicated that catecholamines enhance the ability of nitrosative stress tolerance in *C. jejuni*. In contrast, the *yedYZ*-like operon *Cj0379c-Cj0378c* which play a possible role in the reduction of reactive nitrogen species in the periplasm (Hitchcock et al., 2010) was down-regulated by Epi or NE. Notably, a nitrosative stress regulator NssR (*Cj0466*) was reduced 1.9-fold in expression by NE.

#### Virulence-associated genes

The *Cj0125c*, encoding a DksA-like protein which was reported to control expression of virulence genes in pathogenic bacteria (Yun et al., 2008), was up-regulated by Epi or NE. The *Cj0040, Cj1025c*, and *Cj1242*, which were identified as novel virulence factors co-regulated with flagella (Guerry, 2007), were also up-regulated by Epi or NE. The *Cj1583c* gene (a homolog of *sapB*) and the putative trigger factor Tig (*Cj0193c*), that were involved into antimicrobial peptides resistance (Hoang et al., 2012), were up-regulated by Epi or NE.

#### Enzyme metabolism genes

A bifunctional enzyme IspDF (*Cj1607*) involved into the isoprenoid biosynthesis in the nonmevalonate pathway (Gabrielsen et al., 2004) was up-regulated by Epi or NE. The genes downstream of *Cj1607* (*Cj1608* and *Cj1609*) were also up-regulated by Epi. The Pta gene (*Cj0688*) that encodes phosphotransacetylase for acetate production (Wright et al., 2009) was up-regulated by Epi or NE. Genes of HydB (*Cj1266c*) and NikAB (*Cj1584c-Cj1583c*) that were involved into a NiFe-type uptake system to maintain hydrogenase activity (Howlett et al., 2012) were also up-regulated by Epi or NE. In contrast, the *Cj1161c* gene, encoding a putative CopA homolog (Hall et al., 2008), was down-regulated by Epi or NE. Moreover, its downstream genes *Cj1160c* and *Cj1158c* were also down-regulated by Epi or NE.

#### DNA repair and metabolism

The UvrB (*Cj0680c*) and RecA (*Cj1673c*) genes involved into DNA repair mechanisms in *C. jejuni* (Gaasbeek et al., 2009) were up-regulated by Epi or NE. The gene encoding competence factor ComEA (*Cj0011c*), which function as a periplasmic DNA receptor contributing to the natural transformation of *C. jejuni* (Jeon and Zhang, 2007), was up-regulated by Epi. However, the competence factor Cj1211, which plays a critical role in natural transformation in *Campylobacter* (Jeon et al., 2008), was down-regulated by Epi or NE. The Mfd gene (*Cj1085c*), which encodes a transcription-repair coupling factor involved in strand-specific DNA repair and fluoroquinolone resistance (Han et al., 2008), was down-regulated by Epi.

#### Ribosomal protein biosynthesis

The 22 genes of *rpsJ-rplR* (*Cj1708c-Cj1691c*), *rpsI-rplM* (*Cj1479c-Cj1480c*), and *rpsM-rpsK* (*Cj1592-Cj1593*) involved into ribosomal protein biosynthesis were all up-regulated by Epi or NE. Moreover, genes of *rpsU* (*Cj0370*), *rpmB* (*Cj0450c*), *rplO-rpsE* (*Cj1690c-Cj1689c*) increased in expression by Epi and *rpsD* (*Cj1594*) increased in expression by NE.

#### Genes with opposite expression patterns between catecholamines and iron

Notably, the serum-MEMα medium used in this study was regarded as iron limited condition. Supplementation of such medium with Epi or NE may enable *C. jejuni* to utilize ferric-catecholamine complex as observed in our previous studies (Zeng et al., 2009; Xu et al., 2010). Thus, presence of Epi or NE in the iron-restricted MEMα medium could facilitate catecholamine-mediated iron uptake, consequently leading to increased iron level in cytoplasm. Therefore, the transcriptome profiling of *C. jejuni* NCTC 11168 upon exposure to Epi or NE in this study was also used to compare with two previous publications which analyzed the transcriptome of the bacteria under iron-replete and iron-limited growth conditions by microarray (Palyada et al., 2004) and RNA-seq (Butcher and Stintzi, 2013) respectively (Table S2). It's not surprising that a wide variety of genes involved in iron-uptake, motility, oxidative stress response, and other functions showed a similar expression profiling between this study (in response to catecholamine treatment) and the other two studies focused on the response to iron availability (Palyada et al., 2004; Butcher and Stintzi, 2013). However, it was notable that the transcriptional profiles of some genes in this study were not completely consistent with previous iron transcriptome studies. In particular, expression of some genes was totally in an opposite pattern. For example, this study demonstrated that the operon *Cj1172c-Cj1169c* encoding a hypothetical protein Cj1172c, a peptidyl-prolyl cis-trans isomerase Ppi (*Cj1171c*), a 50 kDa outer membrane protein precursor Omp50 (*Cj1170c*) and a putative periplasmic protein Cj1169c were all up-regulated by Epi or NE. In contrast, all the 4 genes were down-regulated in iron-replete medium (Palyada et al., 2004; Butcher and Stintzi, 2013). Other genes that were activated by Epi or NE in this study but were repressed by high iron level include *typA* (*Cj0039c*), *thrS* (*Cj0206*), *panB* (*Cj0298c*), *fabG* (*Cj0435*), *aspS* (*Cj0640c*), and *fliP* (*Cj0820c*). Moreover, genes that were down-regulated by Epi or NE in this study but were observed to be activated by iron restriction include *birA* (*Cj0099*), *pyrE* (*Cj0233c*), *Cj0760-Cj0761*, *thiE* (*Cj1081c*), *mfd* (*Cj1085c*), *acpP3* (*Cj1304*), *Cj1435c-Cj1436c*, *ctsD* (*Cj1474c*), and *Cj1684c*. This finding strongly suggest that catecholamine-mediated impacts on *Campylobacter* do not solely rely on the iron chelator nature of catecholamines for iron acquisition; catecholamines may also serve as cues to modulate pathobiology of *C. jejuni* through other mechanisms.

## Discussion

In the intestinal lumen, bacteria inevitably encounter the neurotransmitter, in particular, the NE released by noradrenergic neurons of the enteric nervous system (Freestone et al., 2008; Freestone, 2013). The medium chosen to investigate neurotransmitter responsiveness is crucially important in determining the mechanism by which the hormone modulates bacterial growth or virulence (Freestone et al., 2008). The iron-rich broth media such as Luria-Bertani (LB) and tryptic soy broth (TSB) have been used to investigate global effects of catecholamines on gene expression of *Escherichia coli* (Bansal et al., 2007), *Salmonella enteric* (Karavolos et al., 2008), and *Actinobacillus pleuropneumoniae* (Li et al., 2012). However, there have been some concerns for using such iron-replete media to examine the effect of catecholamines on systematic gene expression (Freestone et al., 2008). In particular, interactions of catecholamine with ferric iron in iron-rich medium can lead to the generation of oxygen-derived free radicals and subsequent oxidative stress influencing bacterial gene expression (Freestone et al., 2008). Therefore, it is important to choose appropriate growth medium to mimic *in vivo* environment for studying bacterial response to intestinal catecholamine hormones (Freestone, 2013). In other organisms, the serum-SAPI minimal medium has been used in microarray analysis of *E. coli* (Dowd, 2007) and *S. enterica* (Bearson et al., 2008) upon exposure to catecholamines. These iron-restricted, chemically defined media may minimize confounding factors during transcriptome analysis in response to catecholamine hormones. In this study, the MEMα medium containing 10% FBS was used to study *C. jejuni* growth and gene expression in response to Epi or NE. Significant growth promotion of *C. jejuni* by Epi or NE was also observed in this medium. Notably, the MEMα medium has been successfully used for examining *C. jejuni* transcriptome profiling in response to iron availability in previous *C. jejuni* studies (Palyada et al., 2004; Butcher and Stintzi, 2013).

The findings from this study indicate Epi and NE have similar effect on the transcriptome profile of *C. jejuni* grown in MEMα medium, likely due to their structural similarity for chelating iron (Table 1). However, presence of a panel of unique NE-modulated and Epi-modulated genes (Table 1) suggests that these two catecholamines may exert different effects on *C. jejuni* physiology. A recent study observed significant difference on gene expression of *A. pleuropneumoniae* between Epi and NE treatment (Li et al., 2012).

Epi and NE have been found to enhance the growth ability of *C. jejuni* under iron-restricted conditions in this study and previous publications (Cogan et al., 2007; Zeng et al., 2009; Xu et al., 2010). The effects of Epi or NE on bacterial growth promotion is likely due to catecholamine-mediated iron uptake because the catechol moiety in Epi and NE displays high affinity for ferric iron and can compete the ferric iron sequestered by host iron-binding proteins transferrin and lactoferrin (Freestone et al., 2000, 2008; Lyte, 2004). Subsequently, the ferric-catecholamine complex could be transported into intracellular via iron acquisition system for utilization. An early study suggested that growth response of *E. coli* to NE requires a functional Ent uptake system (Burton et al., 2002). Our previous studies indicated that FeEnt receptor CfrA (but not CfrB) is involved in NE utilization by *C. jejuni* (Zeng et al., 2009; Xu et al., 2010). As expected, most iron uptake players including CfrA, ChuA, and Cj0178 were down-regulated in the presence of Epi or NE. However, we cannot rule out the possibility that catecholamines may enhance bacterial growth and/or pathobiology features through other mechanisms.

To better understand the mechanisms by which bacteria sense catecholamines, the holistic approach microarray has been widely used. In recent studies, global transcriptome profiling of bacteria in response to catecholamines have been analyzed in *E. coli* (Bansal et al., 2007; Dowd, 2007; Kendall et al., 2007), *S. enterica* (Bearson et al., 2008; Karavolos et al., 2008), and *A. pleuropneumoniae* (Li et al., 2012). The catecholamine-modulated genes in other bacteria include those involved in the regulation of various physiological properties (Bansal et al., 2007; Dowd, 2007; Kendall et al., 2007; Bearson et al., 2008; Karavolos et al., 2008; Li et al., 2012). Similarly, in this study, we also observed that similar genes were modulated by Epi or NE, such as those involved in iron uptake, motility, stress response, and other functions.

In this study, we took advantage of previous iron transcriptome data (Palyada et al., 2004; Butcher and Stintzi, 2013) for comparative analysis (Table S2). We observed that almost all the genes involved in iron uptake and motility showed the similar expression patterns between catecholamines and iron though differentially expressed levels exist with some genes. For example, the differentially expressed genes activated by Epi or NE were observed in the flagella assembly components including hook-filament junction, hook, rod and chaperones (Table 4) while almost all the components of the flagellar assembly pathway were up-regulated under iron-replete condition (Butcher and Stintzi, 2013). Comparative analysis of the genes involved in stress response also showed the similar results. In previous studies, the genes involved in oxidative stress defense were repressed by iron, which include *katA, Cj1386, perR, ahpC, trxB*, and *tpx* (Van Vliet et al., 1999; Palyada et al., 2004, 2009; Holmes et al., 2005; Butcher and Stintzi, 2013). Similarly, the genes of *katA, perR*, and *ahpC* were also down-regulated by inactivation of the transcriptional regulator Cj1556, which plays a role in the oxidative and aerobic stress response in *C. jejuni* (Gundogdu et al., 2011). The similar expression profiles of *katA* and *Cj1386* were observed in this study. Moreover, the three enzymes (NrfH-NrfA, NapA/B, and ccoNOQ) involved into nitrosative stress response in *C. jejuni* were increased in expression in response to Epi or NE, similar to transcriptional profiling of the three enzymes in *C. jejuni* during cecal colonization *in vivo* (Woodall et al., 2005) as well as the previous reports on induction of NrfH-NrfA and NapA/B expression by iron (Palyada et al., 2004; Holmes et al., 2005).

Notably, according to comparative analysis of transcriptome data (Table S2), we also observed a panel of genes were uniquely regulated by the presence of NE or Epi in *C. jejuni*, further supporting a hypothesis that catecholamine-mediated iron acquisition is not the only mechanism by which catecholamines influence on the bacterial pathobiology (Freestone et al., 2008). In *E. coli* (Sperandio et al., 2003) and *S*. Typhimurium (Bearson and Bearson, 2008), other regulatory factors have been observed to be involved in the interaction between bacteria and catecholamine hormones. The QseC and QseE sensor kinases have been identified as bacterial adrenergic receptors for catecholamines in *E. coli* (Clarke et al., 2006; Reading et al., 2009). Both the sensors belong to the two-component regulatory systems (QseBC and QseEF) and play a role in the catecholamine-mediated bacterial pathogenesis (Hughes et al., 2009; Reading et al., 2009). The two-component systems QseBC and QseEF also have been proposed to be catecholamine receptors in *S*. Typhimurium (Moreira and Sperandio, 2012; Karavolos et al., 2013). In this study, the *C. jejuni* two-component regulator gene *Cj1608* was observed to be up-regulated 2.1 and 2.3-fold by Epi and NE, respectively. In addition, a putative two-component sensor kinase gene *Cj1492c* was down-regulated 1.6 and 2.1-fold by Epi and NE, respectively (Table S2). Specific role of these regulators in *C. jejuni*-catecholamine interaction needs to be examined in the future.

In this study, some genes showed opposite expression pattern when compared to previous iron transcriptome studies (Palyada et al., 2004; Butcher and Stintzi, 2013); examination of these genes was also highly warranted in the future. For example, among these specific genes, the Omp50 has been characterized as a species-specific outer membrane monomeric porin (Bolla et al., 2000). Recent *C. jejuni* work has demonstrated that Omp50 is a tyrosine phosphorylated kinase that controls capsule polysaccharide biosynthesis or export, and involves in bacterial motility, invasiveness and oxidative stress response (Corcionivoschi et al., 2012). Interestingly, treatment of *C. jejuni* with erythromycin increased expression of Omp50, which represents an adaptive mechanism by reducing cell permeability to Ery (Xia et al., 2013). The enhanced expression of Omp50 in response to NE and Epi observed in this study may have a significant implication in *C. jejuni* infection in the intestine.

## Author contributions

FX, and JL contributed with the conception and design of the study. FX, CW, FG, GC, XZ, BY, and JL were involved in the collection, assembly, analysis and interpretation of data; drafting and revision of the article; and final approval of the article.

### Conflict of interest statement

The authors declare that the research was conducted in the absence of any commercial or financial relationships that could be construed as a potential conflict of interest.
