# Supplementary material for: Transcriptomic analysis of Campylobacter jejuni NCTC 11168 in response to epinephrine and norepinephrine
Source: Front Microbiol. 2015 May 18;6:452. doi: 10.3389/fmicb.2015.00452 (PMC4435418; doi:10.3389/fmicb.2015.00452)
Supplement: Supplementary file 1 [file Table1.DOC]

**Table S1.** Primers used in the qRT-PCR

| Primer | DNA Sequence (5’-3’) |
| --- | --- |
| flgLF | GCTGCAAGCGATAGCAATTC |
| flgLR | CCCGCAAAAAGATACTGACC |
| flgHF | TAGAAAGTGCACCAGGATCG |
| flgHR | AGCTTGTGTGCTTTGAGTGG |
| cj1729cF | AGCAGCTTATTGGGATGCTG |
| cj1729cR | GCAGCAAAAGTTCCACTTCC |
| fdxAF | ATGCGTTGAATGCGTAGGAC |
| fdxAR | CGACTTGGTTGTCCTGATTC |
| cj0037cF | GTATTTTGCCTCACCGCAAC |
| cj0037cR | TCTCCTGGGAAGTGATTTGG |
| chuBF | ATCGGCATAGCAGCTTCATC |
| chuBR | TCCCACACGAAACCAAGAAG |
| cfrAF | ATCAGTTTGCGCCATTGGTC |
| cfrAR | CCAGAAGCACTAACGATTGAGC |
| exbB1F | ATGGCTTGAGTTTGCTTGCG |
| exbB1R | TCAGCTATACCAGGCGCAAC |
| cj0178F | TTTGTAACTCCTGGGGCAAC |
| cj0178R | TGCCTTGAGCTTGATCTGTG |
| cj0131F | GGCAAAACAGGAACAAGTGG |
| cj0131R | CATTGTTCAGGACGCGTTTC |
